# Supplementary material for: Non-Invasive Wildlife Disease Surveillance Using Real Time PCR Assays: The Case of the Endangered Galemys pyrenaicus Populations from the Central System Mountains (Extremadura, Spain)
Source: Animals (Basel). 2023 Mar 23;13(7):1136. doi: 10.3390/ani13071136 (PMC10093302; doi:10.3390/ani13071136)
Supplement: Supplementary file 1 [file animals-13-01136-s001.zip › animals-2274553-supplementary.pdf]

Supplementary material

Tables S1: Real time PCR Target species (validated and "in silico" according to manufacturers (personal communication))

Targeted species	Range of target species	
	Validated with specific template DNA	*in silico* assayed
Leptospira spp.	*L. bratislava (Jez Bratislava)*<br>*L. hardjo (Hardjoprajitno and Hardjo Bovis Sponselee),*<br>*L. canicola (Hond Utrecht IV)*<br>*L. pomona (Pomona)*<br>*L. grippotyphosa (Moskva)*<br>*L. icterohaemorrhagiae (RGA)*<br>*L. autumnalis (Akiyami A)*<br>*L. castellonis (Castellón 3).*	*Leptospira interrogans*<br>*Leptospira kirschneri*<br>*Leptospira noguchii*<br>*Leptospira alexanderi*<br>*Leptospira weilii*<br>*Leptospira borgpetersenii*<br>*Leptospira santarosai*<br>*Leptospira kmetyi*<br>*Leptospira mayottensis*
Listeria spp.	*Listeria monocytogenes,*<br>*Listeria innocua,*<br>*Listeria weishimeri,*<br>*Listeria seeligeri,*<br>*Listeria ivanovii,*<br>*Listeria rocuortidae,*<br>*Listeria marthii.*<br>*Listeria weihenstephanensis*	
Salmonella spp.	*Serovar paratyphi B (CECT 554)*<br>*Serovar enteritidis (CECT 4396) (1,2)*<br>*Serovar typhimurium (CECT 723) (3,4)*<br>*Serovar typhi (CECT 4138)*<br>*Serovar infantis (NCTC 6703)*<br>*Serovar gallinarum (CECT 4182)*<br>*Serovar pullorum (NCTC 10704)*<br>*Serovar choleraesuis (ATCC 10708)*	
Staphilococcus spp.	*S. felis*<br>*S. nepalensis*<br>*S. hominis-5*<br>*S. microti-3*<br>*S. caprae-4*<br>*S. arlettae-5*<br>*S. lentus-6*<br>*S. pasteuri*<br>*S. muscae-4*<br>*S. haemolyticus-4*<br>*S. sciuri-2*<br>*S. chromogenes-8*<br>*S. equorum-1*<br>*S. simulans-5*<br>*S. xylosus*<br>*S. epidermidis-4*	

Targeted species	Range of target species	
	Validated with specific template DNA	*in silico* assayed
	S. aureus-1	
Cryptosporidium spp.	*C. parvum*<br>*C. andersoni*<br>*C. hominis*<br>*C. serpentis*<br>*C. muris*<br>*C. tyzzeri*<br>*C. ubiquitum*<br>*C. ryanae*	*C. apodemi*<br>*C. canis*<br>*C. cuniculus*<br>*C. ditrichi*<br>*C. erinacei*<br>*C. fayeri*<br>*C. galli*<br>*C. hominis TU502*<br>*C. meleagridis*<br>*C. parvum Iowa II*<br>*C. ratti*<br>*C. estudinis*<br>*C. ubiquitum*<br>*C.viatorum*<br>*C.wrairi*
Eimeria spp.	*E. tenella*<br>*E. acervulina*<br>*E. maxima*<br>*E. necatrix*<br>*E. praecox*<br>*E. mitis*<br>*E. brunetti*<br>*E. ovinoidalis*<br>*E. arloingi*<br>*E. ninakohlyakimovae*<br>*E. magna*	*E. acervulina*<br>*E. adenoeides*<br>*E. ahsata*<br>*E. albamensis*<br>*E. arloingi*<br>*E. arnyi*<br>*E. auburnensis*<br>*E. bovis*<br>*E. brunetti*<br>*E.bukidnonensis*<br>*E. canadensis*<br>*E. cf. ictidea*<br>*E. chapmani*<br>*E. coecicola*<br>*E. crandallis*<br>*E. cylindrica*<br>*E. dispersa*<br>*E. ellipsoidalis*<br>*E. exigua*<br>*E. falciformis*<br>*E. flavescens*<br>*E. gallopavonis*<br>*E. granulosa*<br>*E. gruis*<br>*E. intestinalis*<br>*E. irresidua*<br>*E. ivitaensis*<br>*E. kofoidi*<br>*E. lamae*

Targeted species	Range of target species	
	Validated with specific template DNA	*in silico* assayed
		E. legionensis<br>*E. macusaniensis*<br>*E. magna*<br>*E. marsica*<br>*E. maxima*<br>*E. media*<br>*E. meleagrimitis*<br>*E. mitis*<br>*E. necatrix*<br>*E. ovina*<br>*E. pallida*<br>*E. parva*<br>*E. perforans*<br>*E. piriformis*<br>*E. praecox*<br>*E. reichenowi*<br>*E. stiedai*<br>*E. subspherica*<br>*E. tenella*<br>*E. vejdovskyi*<br>*E. weybridgensis*<br>*E. wyomingensis*<br>*E. zuernii*<br>*E. anseris*<br>*E. innocua*<br>*E. meleagridis*<br>***E. caviae***
family ***Ascaridoidea***	Genus *Baylisascaris*<br>Genus *Parascaris*<br>Genus *Ascaris*<br>Genus *Toxascaris*<br>Genus *Toxocara*	

Table S2. Species from digenean orders Plagiorchiida and Opisthorchiida used for in silico primer and probe design of 28S rDNA. NCBI: The National Center for Biotechnology Information

Order	Super- or Family	Genus	Accesion Number from NCBI	Species
Opisthorchiida	Acanthocolpidae	*Monostephanostomum*	EF506763.1	*Monostephanostomum nolani*
Opisthorchiida	Acanthocolpidae	*Neophasis*	MW730773.1-83	*Neophasis oculata*
Opisthorchiida	Acanthocolpidae	*Neophasis*	AF151936.1	*Nephrotrema truncatum*

Order	Super- or Family	Genus	Accesion Number from NCBI	Species
Opisthorchiida	Acanthocolpidae	*Pleorchis*	DQ248215.1	*Pleorchis polyorchis*
Opisthorchiida	Acanthocolpidae	*Pleorchis*	DQ248216.1	*Pleorchis uku*
Opisthorchiida	Acanthocolpidae	*Pseudolepidapedon*	KJ820760.1	*Pseudolepidapedon balistis*
Opisthorchiida	Acanthocolpidae	*Tormopsolus*	DQ248217.1	*Tormopsolus orientalis*
Opisthorchiida	Acanthocolpidae	*Stephanostomum*	AY222256.1; DQ248218.1	*Stephanostomum baccatum*
Opisthorchiida	Acanthocolpidae	*Stephanostomum*	DQ248225.1	*Stephanostomum bicoronatum*
Opisthorchiida	Acanthocolpidae	*Stephanostomum*	DQ248226.1	*Stephanostomum cestillium*
Opisthorchiida	Acanthocolpidae	*Stephanostomum*	DQ248219.1,27	*Stephanostomum cf uku*
Opisthorchiida	Acanthocolpidae	*Stephanostomum*	MW115577.1	*Stephanostomum cf.*
Opisthorchiida	Acanthocolpidae	*Stephanostomum*	DQ248221.1	*Stephanostomum gaidropsori*
Opisthorchiida	Acanthocolpidae	*Stephanostomum*	EF506761.1; MK558796.1	*Stephanostomum sp.*
Opisthorchiida	Cryptogonimidae	*Acanthostomum*	KC489791.1	*Acanthostomum burminis*
Opisthorchiida	Cryptogonimidae	*Acanthostomum*	MK648271.1	*Acanthostomum minimum*
Opisthorchiida	Cryptogonimidae	*Acanthostomum*	KC489792.1	*Acanthostomum sp.*
Opisthorchiida	Cryptogonimidae	*Mitotrema*	AY222229.1	*Mitotrema anthostomatum*
Opisthorchiida	Cryptogonimidae	*Neocladocystis*	MN705808.1	*Neocladocystis bemba*
Opisthorchiida	Cryptogonimidae	*Neocladocystis*	MN705809.1	*Neocladocystis biliaris*
Opisthorchiida	Cryptogonimidae	*Neocladocystis*	MN705810.1	*Neocladocystis sp.*
Opisthorchiida	Cryptogonimidae	*Neocladocystis*	AF300330.1	*Neoglyphe locellus*
Opisthorchiida	Cryptogonimidae	*Stemmatostoma*	MN688143.1	*Stemmatostoma sp.*
Opisthorchiida	Opisthorchiidae	*Clonorchis*	MK450523.1-27; MF099784.1; JF823989.1	*Clonorchis sinensis*
Opisthorchiida	Opisthorchiidae	*Metorchis*	MK482051.1-55	*Metorchis orientalis*

Order	Super- or Family	Genus	Accesion Number from NCBI	Species
Opisthorchiida	Opisthorchiidae	*Metorchis*	KY075772.1-77	*Metorchis sp.*
Opisthorchiida	Heterophyidae	*Cryptocotyle*	MH025622.1-23	*Cryptocotyle lata*
Opisthorchiida	Heterophyidae	*Cryptocotyle*	MW361240.1; AY222228.1	*Cryptocotyle lingua*
Opisthorchiida	Heterophyidae	*Euryhelmis*	AB521800.1.97,99	*Euryhelmis costaricensis*
Opisthorchiida	Heterophyidae	*Procerovum*	HM004179.1	*Procerovum cheni*
Opisthorchiida	Heterophyidae	*Procerovum*	HM004182.1	*Procerovum varium*
Opisthorchiida	Heterophyidae	*Scaphanocephalus*	MT461356.1; MN160569.1-70	*Scaphanocephalus sp.*
Opisthorchiida	Heterophyidae	*Apophallus*	MG806918.1	*Apophallus zalophi*
Opisthorchiida	Heterophyidae	*Ascocotyle*	MK359080.1	*Ascocotyle cameliae*
Opisthorchiida	Heterophyidae	*Centrocestus*	KY075663.1-65	*Centrocestus formosanus*
Opisthorchiida	Heterophyidae	*Centrocestus*	HQ874609.1	*Centrocestus formosanus*
Opisthorchiida	Heterophyidae	*Centrocestus*	LC599535.1-36	*Centrocestus spp.*
Opisthorchiida	Heterophyidae	*Haplorchis*	MN745941.1; KX815125.1	*Haplorchis pumilio*
Opisthorchiida	Heterophyidae	*Haplorchis*	HM004177.1,92	*Haplorchis yokogawai*
Opisthorchiida	Heterophyidae	*Metagonimoides*	JQ995473.1	*Metagonimoides oregonensis*
Opisthorchiida	Heterophyidae	*Metagonimus*	HQ832633.1	*Metagonimus miyatai*
Opisthorchiida	Heterophyidae	*Metagonimus*	MF407172.1-73	*Metagonimus pusillus*
Opisthorchiida	Heterophyidae	*Metagonimus*	LC599529.1,30,32-34,46-47;LC422950.1	*Metagonimus sp.*
Opisthorchiida	Heterophyidae	*Metagonimus*	KX387456.1-60	*Metagonimus suifunensis*
Opisthorchiida	Heterophyidae	*Metagonimus*	HQ832636.1	*Metagonimus takahashii*

Order	Super- or Family	Genus	Accesion Number from NCBI	Species
Opisthorchiida	Heterophyidae	*Metagonimus*	HQ832639.1	*Metagonimus yokogawai*
Opisthorchiida	Heterophyidae	*Phocitrema*	MG806921.1	*Phocitrema fusiforme*
Opisthorchiida	Opisthorchiidae	*Erschoviorchis*	MK877245.1-49	*Erschoviorchis anuiensis*
Opisthorchiida	Opisthorchiidae	*Opisthorchiidae*	MN726966.1	*Opisthorchiidae gen. sp.*
Opisthorchiida	Opisthorchiidae	*Opisthorchis*	MF099790.1	*Opisthorchis felineus*
Opisthorchiida	Opisthorchiidae	*Opisthorchis*	MF110001.1	*Opisthorchis sp.*
Opisthorchiida	Opisthorchiidae	*Opisthorchis*	JF823990.1; HM004188.1	*Opisthorchis viverrini*
Plagiorchiida	Atractotrematidae	*Isorchis*	MF803157.1	*Isorchis currani*
Plagiorchiida	Auridistomidae	*Auridistomum*	AY116872.1	*Auridistomum chelydrae*
Plagiorchiida	Batrachotrematidae	*Opisthioparorchis*	MZ682029.1	*Opisthioparorchis sp.*
Plagiorchiida	Brachycladiidae	*Brachycladium*	KR703279.1	*Brachycladium goliath*
Plagiorchiida	Brachycladiidae	*Orthosplanchnus*	MF611697.1	*Orientocreadium pseudobagri*
Plagiorchiida	Brachycladiidae	*Orthosplanchnus*	MT153593.1	*Orthosplanchnus arcticus*
Plagiorchiida	Brachycladiidae	*Synthesium*	MN295480.1	*Synthesium delamurei*
Plagiorchiida	Brachycladiidae	*Synthesium*	MN295482.1-86	*Synthesium neotropicale*
Plagiorchiida	Brachycladiidae	*Synthesium*	MN295487.1	*Synthesium pontoporiae*
Plagiorchiida	Brachycladiidae	*Synthesium*	MN295481.1	*Synthesium seymouri*
Plagiorchiida	Brachycladiidae	*Synthesium*	MN295488.1-89,92-94,97-99	*Synthesium tursionis*
Plagiorchiida	Brachycladiidae	*Synthesium*	MN295500.1	*Synthesium tursionis*
Plagiorchiida	Brachycladiidae	*Unclassified*	MT153594.1	*Brachycladiidae gen. sp.*
Plagiorchiida	Brachycladiidae	*Zalophotrema*	AY222255.1	*Zalophotrema hepaticum*
Plagiorchiida	Brachycoeliidae	*Brachycoelium*	AF151935.1	*Brachycoelium salamandrae*
Plagiorchiida	Brachycoeliidae	*Mesocoelium*	AY222277.1	*Mesocoelium sp.*
Plagiorchiida	Brachycoeliidae	*Parabrachycoelium*	HQ165754.1	*Parabrachycoelium longicaecum*
Plagiorchiida	Cephalogonimidae	*Cephalogonimus*	AY222276.1	*Cephalogonimus retusus*
Plagiorchiida	Cephalogonimidae	*Cephalogonimus*	MK986866.1-67	*Cercaria baushii*

Order	Super- or Family	Genus	Accesion Number from NCBI	Species
Plagiorchiida	Cephalogonimidae	*Cephalogonimus*	MK986859.1,63-64	*Cercaria cristatella*
Plagiorchiida	Cephalogonimidae	*Cephalogonimus*	MT216312.1	*Cercaria cristatella*
Plagiorchiida	Cephalogonimidae	*Cephalogonimus*	MK259981.1	*Cercaria nigrospora*
Plagiorchiida	Cephalogonimidae	*Cephalogonimus*	MK986861.1	*Cercaria vorskla*
Plagiorchiida	Choanocotylidae	*Choanocotyle*	MW686389.1, 92-93	*Choanocotyle hobbsi*
Plagiorchiida	Choanocotylidae	*Choanocotyle*	EU196356.1	*Choanocotyle hobbsi*
Plagiorchiida	Choanocotylidae	*Choanocotyle*	AY116865.1	*Choanocotyle hobbsi*
Plagiorchiida	Choanocotylidae	*Choanocotyle*	AY116862.1;EU196358.1	*Choanocotyle nematoides*
Plagiorchiida	Choanocotylidae	*Choanocotyle*	EU196355.1	*Choanocotyle platti*
Plagiorchiida	Collyriclidae	*Collyriclum*	JQ231122.1	*Collyriclum faba*
Plagiorchiida	Cortrematidae	*Cortrema*	KJ700420.1	*Cortrema magnicaudata*
Plagiorchiida	Dicrocoeliidae	*Infidum*	MW317227.1,30	*Infidum infidum*
Plagiorchiida	Dicrocoeliidae	*Infidum*	KU726884.1-85	*Infidum similis*
Plagiorchiida	Haematoloechidae	*Haematoloechus*	KY921598.1	*Gyrabascus sp.*
Plagiorchiida	Haematoloechidae	*Haematoloechus*	AF184251.1	*Haematoloechus abbreviatus*
Plagiorchiida	Haematoloechidae	*Haematoloechus*	AF151934.1	*Haematoloechus asper*
Plagiorchiida	Haematoloechidae	*Haematoloechus*	AF387800.1	*Haematoloechus breviplexus*
Plagiorchiida	Haematoloechidae	*Haematoloechus*	AF387797.1	*Haematoloechus complexus*
Plagiorchiida	Haematoloechidae	*Haematoloechus*	AY222280.1; AF387801.1	*Haematoloechus longiplexus*
Plagiorchiida	Haematoloechidae	*Haematoloechus*	AF387799.1	*Haematoloechus medioplexus*
Plagiorchiida	Haematoloechidae	*Haematoloechus*	MN969620.1	*Haematoloechus sp.*
Plagiorchiida	Haematoloechidae	*Haematoloechus*	AF151916.1	*Haematoloechus variegatus*
Plagiorchiida	Haematoloechidae	*Haematoloechus*	AF387798.1	*Haematoloechus varioplexus*
Plagiorchiida	Haploporidae	*Forticulcita*	MT957804.1-21	*Forticulcita minuta*

Order	Super- or Family	Genus	Accesion Number from NCBI	Species
Plagiorchiida	Haploporidae	*Saccocoelioides*	MG925108.1	*Saccocoelioides elongatus*
Plagiorchiida	Haploporidae	*Saccocoelioides*	MG925112.1	*Saccocoelioides magnus*
Plagiorchiida	Haploporidae	*Dicrogaster*	FJ211261.1	*Dicrogaster contracta*
Plagiorchiida	Haploporidae	*Megasolena*	MH244121.1	*Megasolena hysterospina*
Plagiorchiida	Haploporidae	*Megasolena*	MH244122.1, LC626476.1-77	*Megasolena sp.*
Plagiorchiida	Lecithodendriidae	*Gyrabascus*	MK575195.1	*Gyrabascus oppositus*
Plagiorchiida	Lecithodendriidae	*Loxogenes*	KX712084.1-85	*Longiductotrema tethepae*
Plagiorchiida	Lecithodendriidae	*Loxogenes*	AY220624.1	*Loxogenes macrocirra*
Plagiorchiida	Lecithodendriidae	*Ophiosacculus*	AF480167.1	*Ophiosacculus mehelyi*
Plagiorchiida	Lepocreadiidae	*Tetracerasta*	FJ788494.1	*Tetracerasta blepta*
Plagiorchiida	Liliatrematidae	*Liliatrema*	MT303944.1-45	*Liliatrema skrjabini*
Plagiorchiida	Macroderoididae	*Alloglossidium*	MH041415.1-17	*Alloglossidium hamrumi*
Plagiorchiida	Macroderoididae	*Alloglossidium*	MH041418.1	*Alloglossidium hirudicola*
Plagiorchiida	Macroderoididae	*Alloglossidium*	MH041413.1-14	*Alloglossidium macrobdellensis*
Plagiorchiida	Macroderoididae	*Alloglossidium*	MH041419.1-20	*Alloglossidium schmidti*
Plagiorchiida	Macroderoididae	*Alloglossidium*	MH041421.1-22	*Alloglossidium sp.*
Plagiorchiida	Macroderoididae	*Alloglossidium*	MH041423.1-24	*Alloglossidium turnbulli*
Plagiorchiida	Macroderoididae	*Macroderoides*	HQ680851.1	*Macroderoides flavus*
Plagiorchiida	Macroderoididae	*Macroderoides*	HQ680850.1	*Macroderoides sp.*
Plagiorchiida	Macroderoididae	*Macroderoides*	EU850400.1,03; AF433674.1	*Macroderoides spiniferus*
Plagiorchiida	Macroderoididae	*Macroderoides*	EU850398.1	*Macroderoides texanus*
Plagiorchiida	Macroderoididae	*Macroderoides*	EU850406.1	*Macroderoides trilobatus*
Plagiorchiida	Macroderoididae	*Macroderoides*	HQ680846.1; AF433673.1	*Macroderoides typicus*
Plagiorchiida	Microphallidae	*Floridatrema*	AY220632.1	*Floridatrema heardi*
Plagiorchiida	Microphallidae	*Levinseniella*	MG783585.1	*Levinseniella sp.*
Plagiorchiida	Microphallidae	*Levinseniella*	KY752116.1	*Leyogonimus polyoon*

Order	Super- or Family	Genus	Accesion Number from NCBI	Species
Plagiorchiida	Microphallidae	*Microphallidae*	KT355820.1-21; AB974360.1; KX022509.1	*Microphallidae sp.*
Plagiorchiida	Microphallidae	*Atriophallophorus*	MN342153.1-54	*Atriophallophorus winterbourni*
Plagiorchiida	Microphallidae	*Candidotrema*	AY220621.1	*Candidotrema loossi*
Plagiorchiida	Microphallidae	*Maritrema*	AY220629.1	*Maritrema arenaria*
Plagiorchiida	Microphallidae	*Maritrema*	KT355818.1	*Maritrema brevisacciferum*
Plagiorchiida	Microphallidae	*Maritrema*	KT880221.1-23	*Maritrema corai*
Plagiorchiida	Microphallidae	*Maritrema*	KJ144173.1	*Maritrema deblocki*
Plagiorchiida	Microphallidae	*Maritrema*	JF826247.1	*Maritrema eroliae*
Plagiorchiida	Microphallidae	*Maritrema*	MT328208.1-9	*Maritrema kostadinovae*
Plagiorchiida	Microphallidae	*Maritrema*	KJ144178.1	*Maritrema novaezealandense*
Plagiorchiida	Microphallidae	*Maritrema*	AY220630.1	*Maritrema oocysta*
Plagiorchiida	Microphallidae	*Maritrema*	KJ144174.1	*Maritrema poulini*
Plagiorchiida	Microphallidae	*Maritrema*	AY220631.1	*Maritrema prosthometra*
Plagiorchiida	Microphallidae	*Maritrema*	MH257771.1	*Maritrema sp.*
Plagiorchiida	Microphallidae	*Maritrema*	HM584135.1; AF151926.1	*Maritrema subdolum*
Plagiorchiida	Microphallidae	*Microphallus*	AY220626.1	*Microphallus abortivus*
Plagiorchiida	Microphallidae	*Microphallus*	AY220628.1	*Microphallus basodactylophallus*
Plagiorchiida	Microphallidae	*Microphallus*	HM584125.1	*Microphallus calidris*
Plagiorchiida	Microphallidae	*Microphallus*	AY220633.1	*Microphallus fusiformis*
Plagiorchiida	Microphallidae	*Microphallus*	KT355822.1-23	*Microphallus minutus*
Plagiorchiida	Microphallidae	*Microphallus*	MG783586.1-89	*Microphallus ochotensis*
Plagiorchiida	Microphallidae	*Microphallus*	HM584122.1	*Microphallus piriformes*
Plagiorchiida	Microphallidae	*Microphallus*	AY220627.1	*Microphallus primas*
Plagiorchiida	Microphallidae	*Microphallus*	AY220625.1; HM584137.1-38	*Microphallus similis*
Plagiorchiida	Microphallidae	*Microphallus*	HM584131.1,40,42; KJ868216.1	*Microphallus sp.*
Plagiorchiida	Microphallidae	*Microphallus*	HM584139.1	*Microphallus triangulatus*
Plagiorchiida	Monorchiidae	*Palaeorchis*	MT103407.1-10	*Palaeorchis incognitus*
Plagiorchiida	Nanophyetidae	*Nanophyetus*	LT796169.1-70	*Nanophyetus japonensis*
Plagiorchiida	Nanophyetidae	*Nanophyetus*	LN871822.1-23	*Nanophyetus salmincola*
Plagiorchiida	Nanophyetidae	*Nanophyetus*	AY116873.1	*Nanophyetus salminicola*

Order	Super- or Family	Genus	Accesion Number from NCBI	Species
Plagiorchiida	Nanophyetidae	*Nanophyetus*	MG966187.1; LN871818.2,21	*Nanophyetus schikhobalowi*
Plagiorchiida	Nanophyetidae	*Skrjabinophyetus*	AF184252.1	*Skrjabinophyetus neomidis*
Plagiorchiida	Omphalometridae	*Omphalometra*	AF300333.1	*Omphalometra flexuosa*
Plagiorchiida	Omphalometridae	***Rubenstrema***	MK585231.1; AY222275.1; AF300331.1	*Rubenstrema exasperatum*
Plagiorchiida	Orientocreadiidae	*Orientocreadium*	MN969621.1	*Orientocreadium indicum*
Plagiorchiida	Pachypsolidae	*Pachypsolus*	AY222274.1	*Pachypsolus irroratus*
Plagiorchiida	Plagiorchiida	*Aptorchis*	EU334369.1	*Aptorchis aequalis*
Plagiorchiida	Plagiorchiida	*Aptorchis*	EF014729.1	*Aptorchis aequalis*
Plagiorchiida	Plagiorchiida	*Aptorchis*	EF014730.1	*Aptorchis megacetabulus*
Plagiorchiida	Plagiorchiida	*Aptorchis*	EF014727.1	*Aptorchis megapharynx*
Plagiorchiida	Plagiorchiida	*Aptorchis*	EF014728.1	*Aptorchis pearsoni*
Plagiorchiida	Plagiorchiida	*Aptorchis*	AM932523.1	*Aptorchis sp.*
Plagiorchiida	Plagiorchiidae	*Astiotrema*	AF184253.1	*Astiotrema monticellii*
Plagiorchiida	Plagiorchiidae	*Choledocystus*	AY875679.1; HM137617.1	*Choledocystus hepatica*
Plagiorchiida	Plagiorchiidae	*Choledocystus*	MK648310.1	*Choledocystus sp.*
Plagiorchiida	Plagiorchiidae	*Haplometra*	AF151933.1	*Haplometra cylindracea*
Plagiorchiida	Plagiorchiidae	*Macrodera*	MK585199.1; AF151913.1	*Macrodera longicollis*
Plagiorchiida	Plagiorchiidae	*Metaleptophallus*	AF151912.1	*Metaleptophallus gracillimus*
Plagiorchiida	Plagiorchiidae	***Neoglyphe***	AF300329.1; AF300330.1	*Neoglyphe sobolevi*
Plagiorchiida	Plagiorchiidae	***Neoglyphe***	MW730784.1-91	*Neophasis anarrhichae*
Plagiorchiida	Plagiorchiidae	*Paralepoderma*	AF151910.1; MK585218.1	*Paralepoderma cloacicola*
Plagiorchiida	Plagiorchiidae	*Plagiorchiidae*	MK321658.1,72	*Plagiorchiidae sp.*
Plagiorchiida	Plagiorchiidae	*Plagiorchis*	MW528601.1; KJ533392.1-93; AF151911.1; KF556678.1	*Plagiorchis elegans*
Plagiorchiida	Plagiorchiidae	*Plagiorchis*	KJ533394.1; AF151930.1	*Plagiorchis koreanus*

Order	Super- or Family	Genus	Accesion Number from NCBI	Species
Plagiorchiida	Plagiorchiidae	*Plagiorchis*	MW528602.1; KJ533395.1; MK641807.1	*Plagiorchis maculosus*
Plagiorchiida	Plagiorchiidae	*Plagiorchis*	MW528603.1; AF184250.1	*Plagiorchis muelleri*
Plagiorchiida	Plagiorchiidae	*Plagiorchis*	KJ533397.1	*Plagiorchis neomidis*
Plagiorchiida	Plagiorchiidae	*Plagiorchis*	MW528604.1-14,16-26; KY513159.1-62,66,68-74; KJ533398.1; LC599522.1-25	*Plagiorchis sp.*
Plagiorchiida	Plagiorchiidae	*Plagiorchis*	AF151931.1	*Plagiorchis vespertilionis*
Plagiorchiida	Plagiorchiidae	*Skrjabinoeces*	AY222279.1	*Skrjabinoeces similis*
Plagiorchiida	Plagiorchiidae	*Glypthelmins*	AY875678.1	*Glypthelmins poncedeleoni*
Plagiorchiida	Plagiorchiidae	*Glypthelmins*	MN969622.1-23	*Glypthelmins sp.*
Plagiorchiida	Plagiorchiidae	*Leptophallus*	OL675865.1; AF151914.1	*Leptophallus nigrovenosus*
Plagiorchiida	Plagiorchiidae	*Rauschiella*	HM137620.1; AY875677.1	*Rauschiella tineri*
Plagiorchiida	Plagiorchiidae	*Sigmapera*	EF411200.1	*Sigmapera cincta*
Plagiorchiida	Plagiorchiidae	*Travtrema*	MW317228.1-29	*Travtrema stenocotyle*
Plagiorchiida	Pleurogenidae	*Collyricloides*	KP682451.1	*Collyricloides massanae*
Plagiorchiida	Pleurogenidae	*Parabascus*	AY220618.1; MN726967.1	*Parabascus duboisi*
Plagiorchiida	Pleurogenidae	*Parabascus*	AY220619.1	*Parabascus joannae*
Plagiorchiida	Pleurogenidae	*Parabascus*	AF151923.1	*Parabascus semisquamosus*
Plagiorchiida	Pleurogenidae	*Pleurogenidae*	MN726968.1-70	*Pleurogenidae gen. sp.*
Plagiorchiida	Pleurogenidae	*Pleurogenoides*	AF433670.1	*Pleurogenoides medians*
Plagiorchiida	Pleurogenidae	*Prosotocus*	AY220623.1	*Prosotocus confusus*
Plagiorchiida	Pleurogenidae	*Allassogonoporus*	AF151924.1	*Allassogonoporus amphoraeformis*
Plagiorchiida	Pleurogenidae	*Pleurogenes*	AF151925.1	*Pleurogenes claviger*
Plagiorchiida	Pleurogenidae	*Pleurogenes*	MK342571.1	*Pleurogenes sp.*
Plagiorchiida	Pleurogenidae;	*Brandesia*	AY220622.1	*Brandesia turgida*
Plagiorchiida	Pleurogenidae;	*Brandesia*	MT260148.1	*Brandesia turgida*
Plagiorchiida	Prosthogonimidae	*Prosthogonimus*	AY220634.1	*Prosthogonimus cuneatus*

Order	Super- or Family	Genus	Accesion Number from NCBI	Species
Plagiorchiida	Prosthogonimidae	*Prosthogonimus*	AF151928.1; MW114967.1-70; MN726971.1-75	*Prosthogonimus ovatus*
Plagiorchiida	Prosthogonimidae	*Schistogonimus*	AY116869.1	*Schistogonimus rarus*
Plagiorchiida	Reniferidae	*Lechriorchis*	JF820601.1-03; JF820599.1	*Lechriorchis tygarti*
Plagiorchiida	Reniferidae	*Lechriorchis*	AF151932.1	*Lecithopyge rastellus*
Plagiorchiida	Reniferidae	*Ochetosoma*	AF433671.1	*Ochetosoma kansense*
Plagiorchiida	Reniferidae	*Renifer*	HQ665459.1	*Renifer aniarum*
Plagiorchiida	Reniferidae	*Renifer*	LC557508.1-12	*Renifer kansensis*
Plagiorchiida	Reniferidae	*Dasymetra*	AF433672.1	*Dasymetra nicolli*
Plagiorchiida	Stomylotrematidae	*Stomylotrema*	KY982863.1	*Stomylotrema vicarium*
Plagiorchiida	Telorchiidae	*Opisthioglyphe*	MK585341.1-46; AF151929.1	*Opisthioglyphe ranae*
Plagiorchiida	Telorchiidae	*Telorchis*	AF151915.1	*Telorchis assula*
Plagiorchiida	Telorchiidae	*Telorchis*	JF820592.1	*Telorchis bonnerensis*
Plagiorchiida	Telorchiidae	*Telorchis*	OL960085.1	*Telorchis sp.*
Plagiorchiida	Troglotrematidae	*Paragonimus*	HM172616.1	*Paragonimus harinasutai*
Plagiorchiida	Troglotrematidae	*Paragonimus*	DQ836249.1; HM172615.2,17	*Paragonimus heterotremus*
Plagiorchiida	Troglotrematidae	*Paragonimus*	AY116875.1	*Paragonimus iloktsuenensis*
Plagiorchiida	Troglotrematidae	*Paragonimus*	HQ900670.1	*Paragonimus kellicotti*
Plagiorchiida	Troglotrematidae	*Paragonimus*	HM172618.1	*Paragonimus macrorchis*
Plagiorchiida	Troglotrematidae	*Paragonimus*	HM172619.1	*Paragonimus mexicanus*
Plagiorchiida	Troglotrematidae	*Paragonimus*	HM172620.1	*Paragonimus miyazakii*
Plagiorchiida	Troglotrematidae	*Paragonimus*	HM172621.1	*Paragonimus ohirai*
Plagiorchiida	Troglotrematidae	*Paragonimus*	HM004189.1	*Paragonimus pseudoheterotremus*
Plagiorchiida	Troglotrematidae	*Paragonimus*	JQ322628.1-29	*Paragonimus siamensis*
Plagiorchiida	Troglotrematidae	*Paragonimus*	AY116874.1; DQ836244.1,47; JN656173.1-75,78-79,81	*Paragonimus westermani*
Plagiorchiida	Troglotrematidae	*Troglotrema*	MW404388.1	*Troglotrema acutum*
Plagiorchiida	Troglotrematidae	*Troglotrematidae*	AB521803.1	*Troglotrematidae sp.*
Plagiorchiida	Unclassifed	*Xiphidiata*	LC600287.1	*Xiphidiata sp.*

Order	Super- or Family	Genus	Accesion Number from NCBI	Species
Plagiorchiida	Urotrematidae	*Urotrema*	MK477547.1	*Urotrema minuta*
Plagiorchiida	Urotrematidae	*Urotrema*	MK477545.1-46	*Urotrema scabridum*
Plagiorchiida	Urotrematidae	*Urotrema*	MK477548.1-49	*Urotrema shirleyae*
Plagiorchiida	Lissorchiidae	*Lissorchis*	MT928353.1	*Lissorchis cf. gullaris*
Plagiorchiida	Lissorchiidae	*Lissorchis*	MT928354.1	*Lissorchis cf. nelsoni*
Plagiorchiida	Lissorchiidae	*Lissorchis*	EF032689.1; AY222250.1, MT928329.1	*Lissorchis kritskyi*
Plagiorchiida	Macroderoididae	*Paramacroderoides*	MH041375.1	*Paramacroderoides echinus*
Plagiorchiida	Macroderoididae	*Paramacroderoides*	HM137661.1,64	*Paramacroderoides kinsellai*
Plagiorchiida	Macroderoididae	*Perezitrema*	KU535686.1	*Perezitrema bychowskyi*
Unclassified	Unclassified	Trematoda	MH094412.1,13-15,19-23,32,34; LC599542.1	*Trematoda sp.*
